# Preprocessing pupil size data: Guidelines and code

**DOI:** 10.3758/s13428-018-1075-y

**Published:** 2018-07-10

**Authors:** Mariska E. Kret, Elio E. Sjak-Shie

**Affiliations:** 1 Cognitive Psychology Department, Leiden University, Wassenaarseweg 52, 2333 AK Leiden, The Netherlands; 2 Leiden Institute for Brain and Cognition (LIBC), Leiden, the Netherlands; 3 Leiden Institute of Psychology, Leiden University, Leiden, the Netherlands

**Keywords:** Pupil size, Psychophysiology, Instructions, Manual, Open source code

## Abstract

Pupillometry has been one of the most widely used response systems in psychophysiology. Changes in pupil size can reflect diverse cognitive and emotional states, ranging from arousal, interest and effort to social decisions, but they are also widely used in clinical practice to assess patients’ brain functioning. As a result, research involving pupil size measurements has been reported in practically all psychology, psychiatry, and psychophysiological research journals, and now it has found its way into the primatology literature as well as into more practical applications, such as using pupil size as a measure of fatigue or a safety index during driving. The different systems used for recording pupil size are almost as variable as its applications, and all yield, as with many measurement techniques, a substantial amount of noise in addition to the real pupillometry data. Before analyzing pupil size, it is therefore of crucial importance first to detect this noise and deal with it appropriately, even prior to (if need be) resampling and baseline-correcting the data. In this article we first provide a short review of the literature on pupil size measurements, then we highlight the most important sources of noise and show how these can be detected. Finally, we provide step-by-step guidelines that will help those interested in pupil size to preprocess their data correctly. These guidelines are accompanied by an open source MATLAB script (available at https://github.com/ElioS-S/pupil-size). Given that pupil diameter is easily measured by standard eyetracking technologies and can provide fundamental insights into cognitive and emotional processes, it is hoped that this article will further motivate scholars from different disciplines to study pupil size.

Pupil size is nowadays a measure that has become of interest to a broader public than just cognitive psychologists or clinicians. Most eyetrackers provide users with pupil size, but what is often neglected is that any pupillometry data, independent of what kind of system it was measured with, generally requires preprocessing before it can be properly analyzed statistically. The purpose of the present article is therefore to provide the reader with practical advice and open source code that will help analyze pupil size accurately and appropriately; first, however, we give a summary of the mechanism and some brief historical background about the measure of pupillometry and the value of studying pupil size.

## Background

Pupil dilation is regulated by the sympathetic nervous system and mediated almost exclusively via norepinephrine from the locus coeruleus (through stimulation of *α*-adrenoceptors on the iris dilator muscle and postsynaptic *α*_2_-adrenoceptors within the relatively closely located Edinger–Westphal nucleus, which projects to the ciliary ganglion controlling the dilation of the iris; Yoshitomi, Ito, & Inomata, 1985). This dilation response is distinct from the strong contractions exhibited during the pupillary light reflex, which is mediated by acetylcholine (via the iris sphincter muscle). Therefore, under constant low light levels, pupil size is a reliable and accessible measure of norepinephrine levels (Aston-Jones & Cohen, 2005; Koss, 1986; Nieuwenhuis, Aston-Jones, & Cohen, 2005). Although other neurotransmitters, such as serotonin, are known to influence dilation, these effects are similarly known to be mediated via the locus coeruleus–norepinephrine complex (Yu, Ramage, & Koss, 2004).

The pupil has since long been studied extensively as an index of the level of consciousness in coma patients (Teasdale & Jennett, 1974). But as many different disorders are characterized by an imbalance in the sympathetic and the parasympathetic system, the number of studies incorporating measures of pupil size into clinical investigation is growing. Patients with Parkinson’s disease, for instance, have been shown to exhibit a larger pupil diameter after light adaptation, as well as a reduced amplitude of contraction and a prolonged contraction time during the light reflex (Micieli et al., 1991). Another study demonstrated disruptions in pupil responses during voluntary movement preparation in these patients (Wang, McInnis, Brien, Pari, & Munoz, 2016). Alterations in pupil size and/or pupil response have also been observed in psychiatric disorders and have been proposed as indicators of autonomic dysfunction in autism spectrum disorder (Anderson, Colombo, & Unruh, 2013; Martineau et al., 2011), anxiety or depressive disorders (Bakes, Bradshaw, & Szabadi, 1990; Wehebrink, Koelkebeck, Piest, de Dreu, & Kret, 2018), and schizophrenia (Steinhauer & Hakerem, 1992). These studies have highlighted the potential of using low-cost pupil size measurement for diagnosis or to examine executive function deficits in early stages of the disorders.

**Fig. 1:**
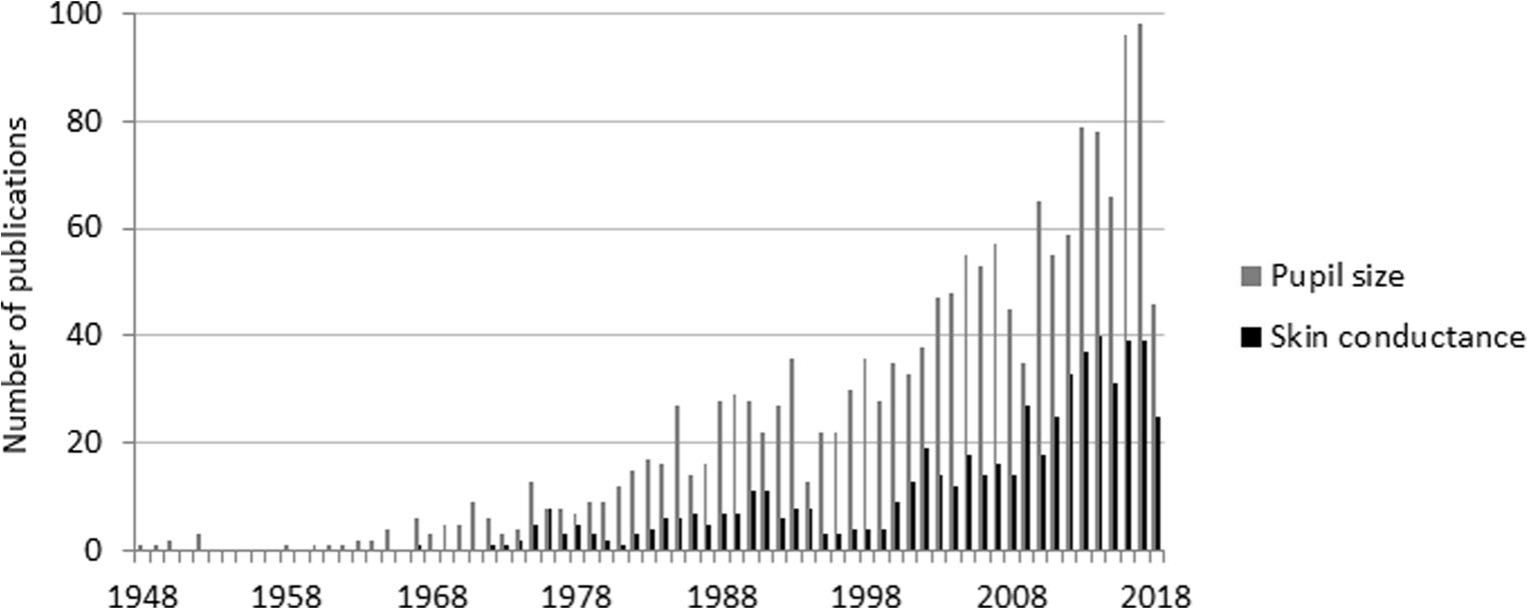
60 years of pupillometry research. A search on PubMed, in June 2018, with the terms [(pupil size[Title/Abstract]) AND eye] yielded a large number of articles published per year (in gray). For a comparison, in black are results for the term (skin conductance response [Title/Abstract]).

The measure of pupil size is a noninvasive indicator of reactions that occur spontaneously during stimulus presentation, do not require overt responses (Laeng, Sirois, & Gredebäck, 2012; Tamietto et al., 2009), and can be observed in infants (Jackson & Sirois, 2009; Wass, de Barbaro, & Clackson, 2015; Wetzel, Buttelmann, Schieler, & Widmann, 2016), patients with psychiatric or neurological disorders (Anderson et al., 2013; Bakes et al., 1990; Martineau et al., 2011; Steinhauer & Hakerem, 1992; Wang et al., 2016), and even nonhuman primates (Iriki, Tanaka, & Iwamura, 1996; Kret, Tomonaga, & Matsuzawa, 2014; Machado, Bliss-Moreau, Platt, & Amaral, 2011; Wang, Boehnke, Itti, & Munoz, 2014; Weiskrantz, Cowey, & Le Mare, 1998). Research during the early years provided evidence that cognitive processes such as problem solving or language comprehension are accompanied by pupil dilation. Figure 1 underscores that ever since the seminal works by Hess and Kahneman (Hess & Polt, 1964; Kahneman & Beatty, 1966), pupillometry has continued to gain popularity in the study of cognition, such that fluctuations have been related to mental arithmetic exercises (Dix & van der Meer, 2015; Klingner, Tversky, & Hanrahan, 2011; Lee, Ojha, Kang, & Lee, 2015), short-term memory (Klingner et al., 2011), and language-processing tasks (Kuipers & Thierry, 2013; Lee et al., 2015; Zellin, Pannekamp, Toepel, & van der Meer, 2011), but more recently also with the study of emotion (Bradley, Miccoli, Escrig, & Lang, 2008; Kinner et al., 2017; Kret, Roelofs, Stekelenburg, & de Gelder, 2013; Kret, Stekelenburg, Roelofs, & de Gelder, 2013; Schrammel, Pannasch, Graupner, Mojzisch, & Velichkovsky, 2009; Tamietto et al., 2009; van Steenbergen, Band, & Hommel, 2011).

In sum, pupillometry rightly has become one of the most widely used response systems in psychophysiology, providing insight into the mechanisms underlying diverse cognitive and affective processes and possible disruptions in clinical groups.

## Guidelines for preprocessing pupillometry data

The purpose of this article is to present a robust and generalizable method for preprocessing pupil size data—that is, for filtering the raw data, removing artifacts, and up-sampling the remaining samples to form a smooth and continuous pupil size time series. The method is designed to work regardless of eyetracker type and sampling frequency, and can be used for a variety of analysis techniques, including multilevel statistics and functional analysis. In addition, the supplied MATLAB code visualizes the applied preprocessing steps, allowing users the effectively review the fitness of the data and filter settings.

The preprocessing pipeline can be broken down into four steps: (1) preparing the raw eyetracker output for processing, (2) filtering the raw data to extract the valid samples subset, (3) up-sampling and smoothing the valid samples, and (4) splitting the data into the relevant segments and analyzing each segment individually. Subsequently, if baseline correction is desired, which is certainly an advisable procedure (Mathôt, Fabius, Van Heusden, & Van der Stigchel, 2018), the output generated by the code can be restructured so that each response value is matched to its baseline value. This allows users to easily apply any desired method for baseline correction.

### Step 1: Preparing the raw data

The first step is to convert the eyetracker output to a standard format containing the raw pupil size time series for the left and/or right eyes, and the signal segmentation information. The latter, generated from the metadata inside the eyetracker output or an auxiliary log file, contains the information necessary to split the recording into the relevant segments. Pupil size samples that are clearly invalid, such as nonpositive pupil size values or samples marked as “invalid” by the eyetracker itself, should be removed at this point as they don’t require specialized filtering.

Because eyetrackers differ in their output format and eyetracking datasets vary in how they should be segmented, performing the abovementioned tasks during the first preprocessing step and generating a standardized data format allows the rest of the pipeline to remain consistent across datasets, with only the settings possibly requiring customization.

### Step 2: Filtering the raw data

**Fig. 2:**
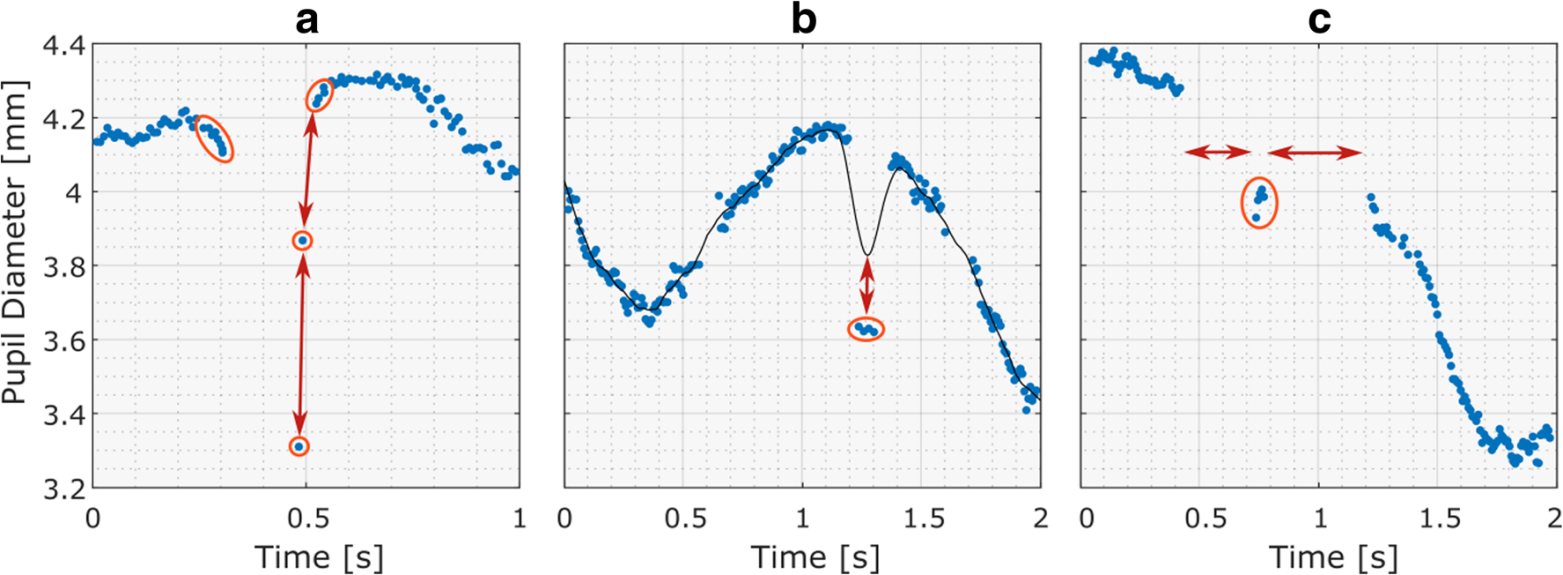
Raw pupil diameter data showing the different kinds of artifacts that are targeted by the raw data filter presented in this article. The invalid samples targeted for rejection are indicated by ovals. (**A**) Certain artifacts, especially those caused by blinks, are characterized by large intersample changes in pupil size—that is, by disproportionately large dilation speeds, as visualized by the arrows. Additionally, the edges of eye-blink gaps may show slopes caused by the onset of eyelid occlusion (see also Fig. 3). (**B**) Outlying clusters of erroneous data points can be identified by their abnormally large deviation from a smooth trend line (solid black line). (**C**) Small islands of spurious samples can be identified by their temporal isolation from other samples, as visualized by the horizontal arrows.

Raw eyetracking data often contain samples that are purely the result of noise or artifacts and therefore carry no useful information for pupil size analysis. Identifying and removing these samples, however, is not a trivial task. This article proposes a filtering pipeline aimed at identifying three types of often-occurring invalid pupil size samples (see Fig. 2): (1) dilation speed outliers and edge artifacts, (2) trend-line deviation outliers, and (3) temporally isolated samples. In addition, pupil size samples that are simply outside of a predefined feasible range, such as between 1.5 and 9 mm when looking at the diameter, can be rejected (e.g., Kret et al., 2014).

Dilation speed outliers are samples that feature a disproportionately large absolute pupil size change relative to their adjacent samples. Because the changes between samples due to actual pupil dilation and constriction are generally less than those resulting from artifacts, such as system errors or blinks, detecting outliers in these changes is an effective way of spotting and rejecting invalid samples. However, due to gaps in the data or nonuniform sampling, it should not be assumed that all data points are equidistantly spaced, nor that all changes between samples are directly comparable. To mitigate this, the absolute change between samples can be divided by the temporal separation of the samples in question, producing the normalized “dilation speed” between samples. Let *d*[*i*] be the pupil size series with corresponding timestamps *t*[*i*]; the dilation speed at each sample (*d'*[*i*]) is calculated as the maximum absolute normalized change relative to either the preceding or the succeeding sample:

1 $$ {d}^{\prime \left[i\right]}=\max \left(\left|\frac{d\left[i\right]-d\left[i-1\right]}{t\left[i\right]-t\left[i-1\right]}\right|,\left|\frac{d\left[i+1\right]-d\left[i\right]}{t\left[i+1\right]-t\left[i\right]}\right|\right). $$

To detect dilation speed outliers, the median absolute deviation (MAD), which is a robust and outlier resilient data dispersion metric (Leys, Ley, Klein, Bernard, & Licata, 2013), is calculated from the dilation speed series, multiplied by a constant (*n*), and summed with the median dilation speed:

2 $$ \mathrm{MAD}=\mathrm{median}\left(\left|{d}^{\prime}\left[i\right]-\mathrm{median}\left({d}^{\prime}\right)\right|\right). $$

3 $$ \mathrm{Treshold}=\mathrm{median}\left({d}^{\prime}\right)+n\bullet \mathrm{MAD}. $$

Samples with dilation speeds above the threshold can now be marked as outliers and rejected.

**Fig. 3:**
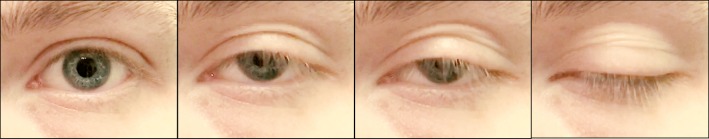
When participants blink, the pupil will momentarily be partly occluded. With some eyetracking systems, this may result in erroneous dips in pupil size (the edge artifacts shown in Fig. 2A).

After the dilation speed outliers have been removed, artifacts around gaps in the data may still remain, especially if these gaps are the result of eye blinks, which may cause pupil size underestimation due to eyelid occlusion (see Fig. 3). Therefore, it is sensible to reject the samples that border certain gaps in the data. Although this is dependent on the eyetracker type used and its pupil detection algorithms, a practical guideline is to reject samples within 50 ms of gaps, with gaps being defined as contiguous missing data sections larger than 75 ms.

Certain eyetrackers, especially those with higher sampling rates, may produce small groups of clearly invalid samples that, since they are clustered together, are resistant to dilation speed filtering. Instead, these invalid samples can be identified by their strong departure from the signal’s trend line, which can be generated by interpolating and smoothing the data that remain after the previous filtering steps. Outliers in absolute trend-line deviations can then be identified and rejected in a similar manner to dilation speed outliers by feeding the absolute trend-line deviations into Eqs. 2 and 3. Subsequently, a new trend line can be generated using the remaining samples, and the outlier detection process can be repeated on all samples considered in the first deviation filter pass. This multipass approach allows for the reintroduction of valid samples that were previously rejected due to the invalid samples “pulling away” the trend line.

Another feature of raw pupil size samples that may indicate invalidity is their sparsity. Since a proper pupil size signal is fairly solid, with continuous gaps during blinks and look-away moments, secluded samples are likely to be the result of noise or a momentary eyetracker glitch, such as erroneous pupil detection during shut eyes. The provided MATLAB code contains a sparsity filter that splits the pupil size signal at the samples that border a gap larger than a first criterion and then rejects the resulting sections that are smaller than a second criterion. Although they are dependent on the dataset, setting these criteria at 40 and 50 ms, respectively, appears to adequately rid the raw eyetracking data of invalid secluded samples.

For the best results, the parameters of the filtering approach introduced in this section, such as *n* in Eq. 3, should be chosen empirically by researchers so that they best fit a particular dataset. It is our experience that no “one size fits all” set of rejection criteria exists, due to differing eyetracker sampling rates, precision, noise susceptibility, and pupil detection algorithms.

### Step 3: Processing the valid samples

At this point, depending on whether monocular or binocular data were collected, one or two valid subsets of the original raw samples remain. If data from both eyes are available, a third “mean pupil size” time series can be generated. Doing so for the time points at which one pupil’s data are missing, however, requires that the dynamic offset between the sizes of the two pupils be taken into consideration. Since the pupil diameters of both eyes are highly correlated, especially locally (Jackson & Sirois, 2009), this dynamic offset can be calculated at the time points that have both pupils’ data, interpolated to the time points at which only a single pupil’s size is available, and used to generate the “mean” pupil size in the presence of missing samples.

**Fig. 4:**
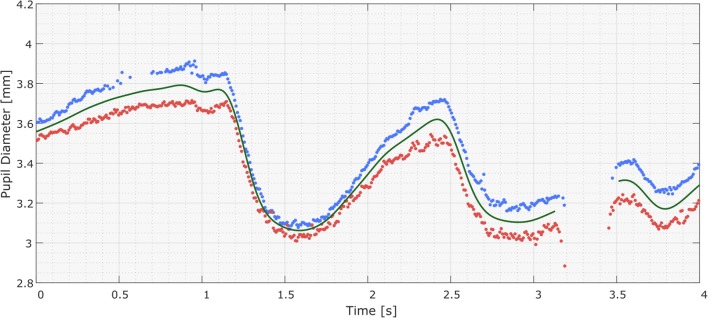
Results of the preprocessing pipeline, showing the raw pupil diameter samples for the right and left eyes (blue and red dots, respectively) and the interpolated and low-pass-filtered “mean pupil diameter” signal (green curves). The interpolated and filtered signals of the left and right pupils are not shown. The mean pupil diameter signal was generated from the valid raw samples of both pupils, including during the absence of one pupil’s data, in which case the local pupil size difference was estimated and used to generate the “mean pupil size” value (as can be seen at 0.6 s). The settings used stipulated that the signals were not to be interpolated over gaps larger than 250 ms—hence, the missing data around 3.3 s.

The left, right, and/or “mean” pupil size time series now consist of nonequidistantly spaced data points, with gaps where data have been removed. To increase the temporal resolution and smoothness of the data, the data points can be resampled with interpolation to a high sampling rate, such as 1000 Hz. The resulting signal can then be smoothed using a zero-phase low-pass filter, with a suggested cutoff frequency of 4 Hz (Jackson & Sirois, 2009). See Fig. 4. Subsequently, sections that were interpolated over unacceptably large gaps can be set to “missing,” as is visualized by the gap in Fig. 4. This filtered signal—which, given a sampling rate of 1000 Hz, has a temporal resolution of 1 ms—can now be summarized for the desired time windows. One can, for instance, calculate the mean pupil diameter for the pre- and poststimulus sections and use these to determine the relative pupil size change or to calculate the mean for short, 100-ms sections within a trial to produce a time series suitable for multilevel statistics (Kret & De Dreu, 2017; Kret, Fischer, & De Dreu, 2015; Kret, Roelofs, et al., 2013; Kret, Stekelenburg, et al., 2013).

In studies with little data available—for instance, in clinical studies or primate research—it can sometimes be preferred to leave the data as untouched as possible. For example, in Kret, Tomonaga, and Matsuzawa (2014), we included each data point, sampled every 16.67 ms (i.e., at 60 Hz) in a multilevel time-course analysis with time points nested within trials, which were nested within sessions nested within test subjects (Kret et al., 2014). In this case, when data from two eyes are recorded, it is even possible to use “eye” as another hierarchical level of analysis.

### Step 4: Data sectioning and analysis

Once the valid raw samples have been interpolated and filtered, the relevant sections within these signals can be analyzed individually using the segments defined in Step 1. Standard descriptive metrics that can be extracted for each section include the mean, maximum, minimum, standard deviation of the pupil diameters, and missing data percentage. The latter measure can be used to reject sections that do not feature enough data.

## Discussion

The study of pupil size is becoming increasingly popular and is one of the most widely used response systems in psychophysiology (Eckstein, Guerra-Carrillo, Miller Singley, & Bunge, 2017; Wang & Munoz, 2015). Pupillary changes can reflect diverse cognitive and emotional states (Harrison, Singer, Rotshtein, Dolan, & Critchley, 2006; Kret & De Dreu, 2017; Kret et al., 2015), and this information can in turn be applied to widely different settings, ranging from clinical practice to traffic safety and consumer psychology. Numerous systems are being used for measuring this signal, ranging from the head-mounted systems that are most popular in the psychology lab, to more flexible, remote systems in infant research, or eyetracking glasses that users can wear while driving on the road or walking around in a shopping mall and scanning various products. Since eyetracking systems can vary considerably in their sampling rate, precision, and noise susceptibility, as well as in the way they mark missing data, we believe it to be of crucial importance to always thoroughly inspect the signals and the efficacy of the preprocessing pipeline prior to analyzing the pupil size data. Indiscriminate inclusion of all available data or the use of nonrobust outlier rejection methods may result in unnecessarily contaminated datasets, which could lead to incorrect interpretations of the pupil size data.

With this article and the accompanying code, we hope to have provided a generalized method for preprocessing raw pupil size data. The presented approach is designed to work regardless of eyetracker specifications and can output summary pupil diameter data for time segments of arbitrary location and duration. This not only makes it suitable for various statistical analysis techniques, but also allows for the synchronized analysis of eyetracking signals and other simultaneously collected data, such as skin conductance or heart rate.

The preprocessing pipeline we have presented focuses mainly on data filtering and smoothing, and can therefore still benefit from the addition of data correction and feature detection functions. First, an often neglected confound in pupil size analysis is the effect of gaze position on the recorded pupil size (Gagl, Hawelka, & Hutzler, 2011), sometimes referred to as the “pupil foreshortening error” (Hayes & Petrov, 2016). When using a standard stationary eyetracking camera and affixed participant head setup, rotations of the eyes change the angle at which the camera records the pupil, and therefore also the pupil’s apparent size. As such, this manifestation of gaze position in pupil size should ideally be controlled or corrected for, which we acknowledge as a current limitation of our preprocessing pipeline and the provided code’s functionality. Similarly, the pupil size is strongly affected by luminance, which cannot always be controlled for in the experimental setting and may mask the responses related to cognitive factors. Other than allowing baseline correction, our preprocessing pipeline does not feature any methods for dealing with varying luminance effects, which can be a limiting factor when attempting to extract a metric of cognitive effects from pupillometry data. However, the up-sampled and smoothed pupil size signals generated by the code, as well as its signal segmentation functionality, may provide a useful starting point for further processing—for example, when performing pupil size deconvolution-based analysis. Finally, the provided code has the limitation that it does not label eye blinks, which is less than optimal since eye blinks could be useful as an index of resting-state dopamine activity and to help identify clinical disorders (Jongkees & Colzato, 2016). Instead, our approach calculates the difference between consecutive pupil size samples but only applies these differences to artifact rejection, passing up on the opportunity to also use them for blink detection (Hershman, Henik, & Cohen, 2018).

All in all, we believe that code provides a valuable addition to the existing literature and gives researchers concrete handles to deal with pupillometry data in an appropriate way. Because the code visualizes the processed data as well as all intermediate filtering steps, it allows researchers to identify the effect of the filter parameters and to optimize them for their particular dataset. Although the provided MATLAB codebase contains all necessary functions and classes to implement the approach presented here, users will still need to modify specific sections of the code to fit their data and analysis needs. Nevertheless, we hope that the included examples provide a helpful overview of the analysis pattern and why certain steps need to be taken. Moreover, we believe that our open source MATLAB code and its modular design provides a valuable and accessible framework for solving common pupil size data preprocessing challenges.
